# Antiviral mode of action of bovine dialyzable leukocyte extract against human immunodeficiency virus type 1 infection

**DOI:** 10.1186/1756-0500-4-474

**Published:** 2011-11-01

**Authors:** Humberto H Lara, Liliana Ixtepan-Turrent, Elsa N Garza-Treviño, Jose I Badillo-Almaraz, Cristina Rodriguez-Padilla

**Affiliations:** 1 Laboratorio de Inmunología y Virología, Departamento de Microbiología e Inmunología, Universidad Autonoma de Nuevo Leon, Nuevo Leon, Mexico

## Abstract

**Background:**

Bovine dialyzable leukocyte extract (bDLE) is derived from immune leukocytes obtained from bovine spleen. DLE has demonstrated to reduce transcription of Human Immunodeficiency Virus Type 1 (HIV-1) and inactivate the nuclear factor kappa-light-chain-enhancer of activated B cells (NF-κB) signaling pathway. Therefore, we decided to clarify the mode of antiviral action of bDLE on the inhibition of HIV-1 infection through a panel of antiviral assays.

**Results:**

The cytotoxicity, HIV-1 inhibition activity, residual infectivity of bDLE in HIV-1, time of addition experiments, fusion inhibition of bDLE for fusogenic cells and the duration of cell protection even after the removal of bDLE were all assessed in order to discover more about the mode of the antiviral action.

HIV-1 infectivity was inhibited by bDLE at doses that were not cytotoxic for HeLa-CD4-LTR-β-gal cells. Pretreatment of HIV-1 with bDLE did not decrease the infectivity of these viral particles. Cell-based fusion assays helped to determine if bDLE could inhibit fusion of Env cells against CD4 cells by membrane fusion and this cell-based fusion was inhibited only when CD4 cells were treated with bDLE. Infection was inhibited in 80% compared with the positive (without EDL) at all viral life cycle stages in the time of addition experiments when bDLE was added at different time points. Finally, a cell-protection assay against HIV-1 infection by bDLE was performed after treating host cells with bDLE for 30 minutes and then removing them from treatment. From 0 to 7 hours after the bDLE was completely removed from the extracellular compartment, HIV-1 was then added to the host cells. The bDLE was found to protect the cells from HIV-1 infection, an effect that was retained for several hours.

**Conclusions:**

bDLE acted as an antiviral compound and prevented host cell infection by HIV-1 at all viral life cycle stages. These cell protection effects lingered for hours after the bDLE was removed. Interestingly, bDLE inhibited fusion of fusogenic cells by acting only on CD4 cells. bDLE had no virucidal effect, but could retain its antiviral effect on target cells after it was removed from the extracellular compartment, protecting the cells from infection for hours.

bDLE, which has no reported side effects or toxicity in clinical trials, should therefore be further studied to determine its potential use as a therapeutic agent in HIV-1 infection therapy, in combination with known antiretrovirals.

## Background

The pandemic of Human Immunodeficiency Virus Type 1 (HIV-1) infection, the cause of Acquired Immunodeficiency Syndrome (AIDS), is a grave public health issue and ranks among the greatest infectious disease scourges in history [1]. There were more than 33.3 million people worldwide with HIV-1 infection or AIDS, according to the latest estimates by the Joint United Nations Program on HIV/AIDS (UNAIDS) [2].

The use of highly active antiretroviral therapies has dramatically reduced morbidity and mortality among patients infected with HIV-1 [3,4]. However, the success of antiretroviral treatment is frequently restricted by the emergence of HIV-1 drug resistance [5]. Therefore, the search for new drugs to inhibit viral replication [6] or to restore the immune system in HIV-1 patients continues. Newly discovered naturally derived or chemically synthesized substances are continuously being evaluated as therapeutic drug candidates with antiviral activity. These potential drugs are eagerly awaited and may prove beneficial for the growing number of HIV-infected individuals who have developed resistance to the currently available antiretrovirals [7].

Dialyzable Leukocyte Extract (DLE) is derived from immune leukocytes and contains low molecular weight proteins (< 10, 000 Da) [8]. DLE possess three chromatographic fractions (Fa, Fb and Fc), Fraction Fb inhibits viral production more than 80%. Therefore, fractions Fa and Fc did not show inhibitory effect for any viral dose used [9].

This preparation is a modulator of the immune response that is able to transmit the ability to express delayed-type hypersensitivity (DTH) and cell mediated immunity (CMI) from sensitized donors to immune deficient recipients [10]. DLE also mediates effects on immune system functions, further influencing its response. These effects include cytokine modulation [11,12], the activation of monocyte and macrophage chemotaxis [13] and natural killer activity enhancement [14]. The therapeutic and prophylactic applications have been the most important and interesting aspects of DLE [15], principally because there has been no reported side effects or toxicity in humans [16].

DLE has demonstrated to be effective in those diseases in which Cell-Mediated Immunity (CMI) plays a relevant role in protection against and control of the disease, such as viral infections ((herpes zoster [17], hepatitis B [18], intracellular bacterial diseases like tuberculosis [19] and leprosy [20], parasite infections, such as leishmaniasis [21] or cryptosporidiosis [22], and fungal infections (mucous-cutaneous candidiasis [23]), as well as in primary immunodeficiencies (Wiskott Aldrich syndrome [24], Behçet's syndrome [25]), bronchial asthma [26], otitis media [27], uveitis [28]) and some types of cancer [29,30].

Previously we reported that bovine DLE (bDLE) was useful as an adjuvant in breast cancer patients undergoing chemotherapy, demonstrating protective effects against myelosuppression secondary to antitumoral drugs by improving cellular and humoral immunity, as well as in regulating the production of different cytokines involved in cellular proliferation [16,29,30]. Furthermore, *in vitro *assays demonstrated that bDLE affected the regulation of the expression of p53, bab-1, c-myc, bax, bcl-2 and bad mRNA [31,32]. Nowadays, the majority of the studies on DLE are limited to diseases that occur with chronic inflammation [33], like HIV-1 infection.

A main feature of HIV infection is the expression of several proinflammatory cytokines expressed as soluble factors or membrane-bound molecules that regulate both HIV replication and T cell apoptosis. Proinflammatory cytokines have key roles in the HIV lifecycle, especially at the level of transcription, by enhancing the ability of HIV to establish latent reservoirs on HIV infected patients. In addition, several HIV proteins, such as Nef, Tat, and Vpr hijack proinflammatory cytokine signaling, further underlining the potential importance of inflammation in HIV pathogenesis. Moreover, an *in vivo *chronic inflammatory state has been correlated to increased levels of viremia and accelerated disease progression [34]. DLE has been used to treat HIV-1 infected patients, either asymptomatic or at the AIDS phase, resulting in a partial immune reconstitution [35,36], a lower incidence of opportunistic infections [37], and clinically relevant improvement [16,38].

Viruses have evolved to modulate the NF-kB pathway to enhance viral replication, improve host cell survival, and evade the immune response [39]. With HIV, viral and cellular membrane fusion activates NF-κB, a process that requires CD4^+ ^T cells. HIV-1 contains regulatory regions in its long terminal repeat (LTR) implicated in the control of viral gene expression that contain three Sp1 core promoter binding sites and two NF-κB core enhancer motifs [40] that are recognized by endogenous host cell transcription factors. These are important regulatory elements in the LTR that control expression of the promoter along with Tat, a viral transactivator protein necessary for HIV-1 replication [41].

Previous studies have reported DLE *in vitro *reduced HIV-1 transcription [42] by regulating activation of NF-κB and Sp1 transcription factors [42-44]. Other studies reported DLE induced the production of leukocytes and reduced TNF-α [44] and TFG-β1 [45] secretion, which are cytokines that play a pivotal role in HIV-1 pathogenesis by up-regulating the transcription of HIV-1 and increasing the expression of HIV co-receptor CXCR4, respectively. Additionally, envelope glycoprotein gp120 can signal NF-κB by engaging the CD receptor in a pathway that involves p56 and activates NF-κB and HIV-1 LTR transcription [46]. HIV-1 gene expression and transcription is an essential step in the viral life cycle and is considered to be a possible target for the inhibition of HIV-1 replication [43]. The exact mechanism of action of bDLE is still unclear, however we focused on the bDLE mode of action against HIV-1 infection.

## Results

### Cytotoxic effect

**Figure 1:**
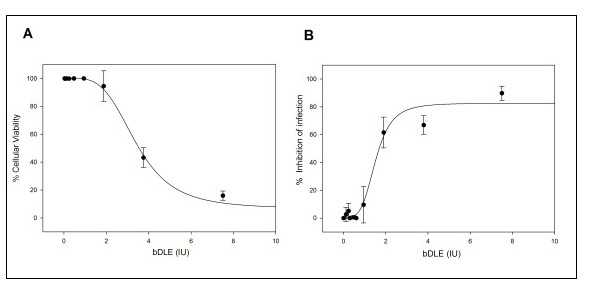
**Cytotoxicity assessment of bDLE and HIV-1 inhibition activity**. **A**) HeLa-CD4-LTR-β-gal cells (5 × 10^4 ^cells/well) and **B**) HIV-1_IIIB _cell-free viruses (MOI 0.2-0.5) were challenged with two-fold serial dilutions of bDLE. Cell viability and β-gal activity were measured with a luciferase-based assay 24 h after nanosilver exposure. Percentage values are relative to the positive control (no compound treatment). The data represent the means ± standard deviations from three separate experiments, each of which was carried out in duplicate.

The half cytotoxic concentration (CC_50_) of bDLE when exposed to HeLa-CD4-LTR-β-gal cells was 3.41 ± 0.1 IU (P < 0.0001) (Figure 1A).

### Range of antiviral activity

bDLE was tested against an HIV-1_IIIB _isolate using indicator cells in which infection was quantified by a luciferase-based assay. The concentration of bDLE at which HIV-1_IIIB _infectivity was inhibited by 50% (IC_50_) was found to be 1.53 ± 0.1 IU (P < 0.0001) (Figure 1B). In addition, bDLE inhibited HIV-1_IIIB _infectivity at doses that were not cytotoxic for HeLa-CD4-LTR-β-gal cells. The therapeutic index (TI = CC_50_/IC_50_) for bDLE in these cells was then calculated to be 2.23. The therapeutic index reflects a compound's overall efficacy by relating cytotoxicity (CC_50_) with effectiveness, measured as the ability to inhibit infection (IC_50_), under the same assay conditions.

### Virucidal activity

**Figure 2:**
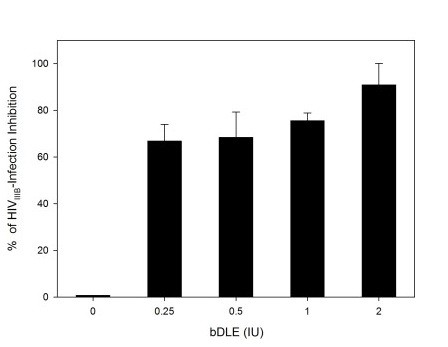
**Residual activity in HIV-1 strains**. HIV-1_IIIB _cell-free viruses were exposed to serial dilutions of bDLE for 5 minutes. The viruses were then ultracentrifuged, washed twice and added to HeLa-CD4-LTR-β-gal cells. After 24 hours, β-gal activity was measured. Percentage values are relative to the positive control (infected cells without bDLE treatment). The data represent the means ± standard deviations from three separate experiments, each of which was carried out in duplicate.

To determine if the bDLE might have effects on the virus itself, HIV-1_IIIB _isolates were treated with different concentrations of bDLE. After removal of bDLE, the residual infectivity of the cell-free viruses was quantified by a luciferase-based assay. As shown in Figure 2, bDLE pretreatment of HIV-1_IIIB _did not decrease the infectivity of the viral particles in a dose dependent manner.

### Inhibition of Env/CD4-mediated membrane fusion

**Figure 3:**
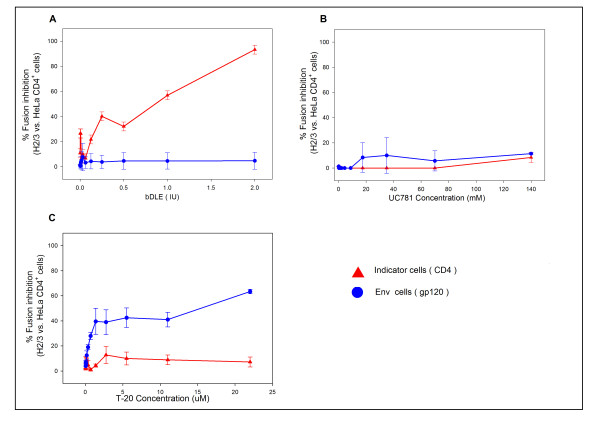
**Inhibition of Env/CD4-mediated membrane fusion**. β-gal activity was measured after CD4 cells and Env cells were co-cultured after exposure to **A**) bDLE, **B**) UC781 and **C**) T-20 under different circumstances: (■) CD4 cells were exposed to the compound and co-cultured with Env cells for 24 hours; (▲) CD4 cells were exposed to the compound for 30 minutes, washed, and co-cultured with Env cells for 24 hours; (✘) Env cells were exposed to the compound for 30 minutes, washed, and co-cultured with CD4 cells for 24 hours. Percentage values are relative to the positive control (cell-to-cell fusion without pretreatment with drug). The data represent the means ± standard deviations from three separate experiments, each of which was carried out in duplicate.

A cell-based fusion assay was used to mimic the gp120-CD4-mediated fusion process of HIV-1 with bDLE. When bDLE was exposed first to the CD4 cells-Env cells mixture, fusion between both cells was blocked in a dose-dependent manner. Cell-based fusion was also inhibited when bDLE was applied only to CD4 cells for 30 minutes, followed by bDLE removal. However, when Env cells were first exposed to bDLE for 30 minutes, then removed and added to CD4 cells, fusion between both cells was not inhibited (Figure 3A). Known antiretroviral drugs, such as UC781 (NNRTI), were used as controls in this cell based fusion assay and did not inhibit cell fusion (Figure 3B). T-20 (Fusion Inhibitor), did inhibit cell fusion in all assays, however, except when exposed to CD4 cells for only 30 minutes and then removed, after which the cells were mixed (Figure 3C).

### Time (Site) of Intervention

**Figure 4:**
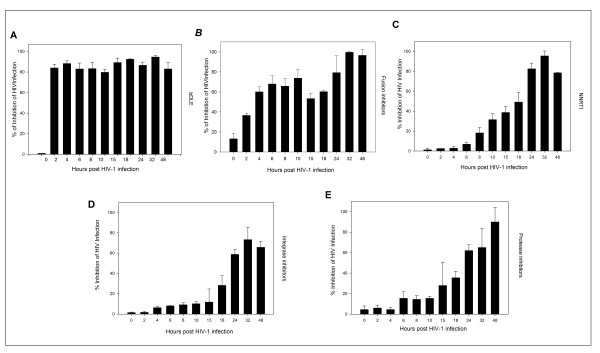
**Time of intervention in HIV-1 life cycle**. HeLa/CD4-LTR-β-gal cells were infected with HIV-1_IIIB _cell-free virus before **A**) bDLE (2 IU), **B**) T-20 (100 μM), **C**) UC781 (70 nM), **D**) 118-D-24 (120 μM) and **E**) Amprenavir (0.1 mM), were added upon HIV-1 inoculation (time zero) or at various time points post-infection. β-gal activity was measured following 24 hr of incubation. Percentage values are relative to the positive control (infected cells without drug pretreatment). The data represent the means ± standard deviations from three separate experiments, each of which was carried out in duplicate.

To further determine the antiviral target of bDLE, a time-of-addition experiment was performed using a single cycle infection assay. The time-of-addition experiment was used to determine the stage(s) of the viral life cycle that were blocked by bDLE. Several antiretroviral drugs were chosen as controls as they mark different stages of the viral cycle (i.e., fusion or entry, retrotranscription, protease activity, and integration into the genome). As seen in Figure 4(A-E), the antiviral activity of T-20, UC781, 118-D-24 and Amprenavir started to decline after the cycle stage that they targeted was passed. The fusion inhibitor's activity declined after 2 h (Figure 4B), the RT inhibitor after 8 h (Figure 4C), the integrase inhibitor after 18 h (Figure 4D) and the protease inhibitor after 15-18 h (Figure 4E). In contrast, bDLE retained its antiviral activity up to 48 h (Figure 4A) after the HIV inoculation, inhibiting HIV-1 infection in 80% of infected cells against the positive control.

### Cell protection assays

**Figure 5:**
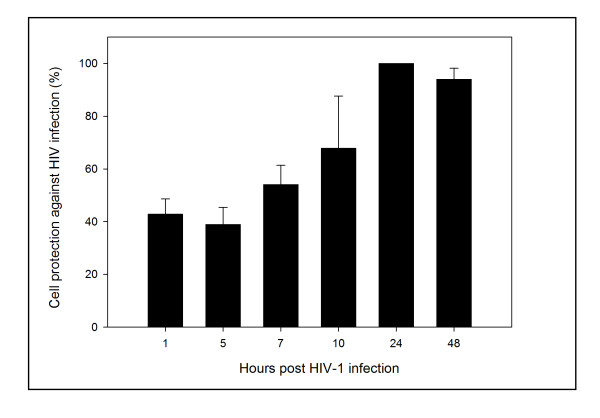
**Cell protection against HIV-1 infection**. β-gal activity was measured after HeLa/CD4-LTR-β-gal cells were exposed to bDLE (2 IU) for 30 minutes, washed and exposed to HIV-1_IIIB _cell-free virus at various time points post-treatment. Percentage values are relative to the positive control (infected cells without drug pretreatment). The data represent the means ± standard deviations from three separate experiments, each of which was carried out in duplicate.

HeLa-CD4-LTR-β-gal cells were pretreated with bDLE for 30 minutes, which was then removed from the extracellular compartment with three washes, and subsequently the HeLa-CD4-LTR-β-gal cells were exposed to HIV-1_IIIB _for different pretreatment times (1, 5, 7, 10, 24 and 48 hours). As shown in Figure 5, HIV-1 infection was inhibited, as host cells were protected even after 7 hours from the bDLE removal.

## Discussion

Due to the DLE immunomodulatory properties discussed before, progression to AIDS in asymptomatic HIV-1 infected individuals treated with conventional anti-retrovirals, has shown a retarded progression to AIDS under adjuvant treatment with dialyzable leukocyte extract (DLE), demonstrated by lower incidences of opportunistic infections and improved cellular immunity [9,38,44]. DLE simultaneously shows anti-HIV activity [44,47,48], modulates different types of immune effectors (*e.g*., cytokines and transcription factors)[11,47,49] and restores leukocyte subsets in treated patients [16,29,35,37]. All these properties make DLE a potential drug to be used in a therapeutic combination with antiretrovirals to improve immune and clinical responses.

Bovine dialyzable leukocyte extract (bDLE) is defined as the dialyzate of a heterogeneous mixture of low molecular weight substances released from disintegrated blood leukocytes or lymphoid tissue obtained from homogenized bovine spleen. Previous studies have shown inhibition of HIV-1 infection by suppression of the activity of essential transcription factors [42-44] and cytokines by DLE. The purpose of this study was to demonstrate the mechanism of antiviral action of bDLE *in vitro *in the inhibition of HIV-1 and the protection of host cells from infection.

First, we compared the half cytotoxic concentration of bDLE when exposed to HeLa-CD4-LTR-β-gal cells (CC_50 _= 3.41 IU) (Figure 1A) with the concentration of bDLE at which HIV-1_IIIB _infectivity was inhibited by 50% (IC_50 _= 1.33 IU) (Figure 1B) [48]. Then, the therapeutic index was determined (TI = 2.56) and used as an indicator of bDLE overall efficacy and safety. Despite the fact that TI was lower than expected (< 10), bDLE is a compound that has been used in clinical assays for more than fifty years without adverse reactions [10]. Better understanding of its inhibition mechanism can contribute to the development of new and improved anti-HIV-1 agents, which could be more efficient and have lower cytotoxicity. Furthermore, the residual infectivity [50,51] of cell-free viruses after bDLE treatment showed no inhibitory activity (Figure 2), which suggests that bDLE does not act as a virucide on the viral membrane to inhibit infection. bDLE showed inhibition of fusogenic cell-cell interactions in a dose-dependent manner when bDLE was exposed to CD4 cells, bDLE was then removed and the CD4 cells were mixed with Env cells. However, after 30 minutes pretreatment of Env cells with bDLE and then transfer to CD4 cells, there was no inhibition of Env-CD4 cell fusion. This observation further supported our previous results that bDLE acted on CD4 expressing cells and not on the Env of the HIV-1 virus. Furthermore, when exposed to CD4 cells for only 30 minutes, which is the time required for conformational changes in gp120 after CD4 binding [52], bDLE again showed inhibition in a dose-dependent manner (Figure 3A). These data suggested that bDLE acted on CD4 cells to inhibit HIV-1 infection. Antiviral results reported by Fernandez-Ortega *et al*. [53,54] have also contributed to the knowledge of the molecular mechanisms responsible for the effectiveness of bDLE against HIV infection.

To further determine the antiviral target of bDLE, a time-of-addition experiment was used to define the stage(s) of the viral life cycle that are blocked by these compounds. These results were compared with several antiretroviral drugs as controls that marked different stages of the viral cycle [55-61] (Figure 4B-E). Our findings suggested that bDLE highly inhibited HIV-1 infection at all stages (Figure 4A), possibly due to viral Tat protein down-regulation (Tat activates β-galactosidase indicator gene expression in HeLa-CD4-LTR-β-gal cells). Inhibition of HIV-1 Tat activity correlates with down-regulation of *bcl*-2 [62], but the action of bDLE on *bcl*-2 has not yet been determined in HIV-1 studies. Previously, *bcl*-2 was found to be reduced in breast cancer cell lines when treated with bDLE [32]. Furthermore, DLE inactivated the NF-κB signaling pathway by reducing the secretion of cytokines, such as IL-1 and TNF-α, which are effective inducers of NF-κB activity [63]. In HIV-infected T cells, NF-κB-dependent transactivation is essential for HIV-LTR induction. Interestingly, even the function of HIV Tat in resting CD4 T lymphocytes depends on κB responsive elements in the LTR [46]. Based on these interesting findings, it will be necessary to focus specifically on the transcriptional factors (NF-κB and SP1) and pro-apoptotic genes (*bcl*-2) in future research on bDLE as an antiviral against HIV-1 infection.

Lastly, bDLE was capable of rendering CD4 expressing cells resistant against HIV-1 infection by residual active virus for several hours [64]. Previous results indicated that, although pretreatment of cells (MT-4) with DLE for 3 hours had no effect, inhibition of HIV-1 production was observed when cells were pre-treated for a longer period of time (from 1 to 7 days), an effect that was characterized by the decline in TNFα and TGFβ1 gene expression and inhibition of transcriptional factors [9]. In our assays, after pretreatment of the HeLa-CD4-LTR-β-gal cells and bDLE removal prior to viral challenge, protection against infection lasted 7 hours after bDLE was removed from the extracellular compartment (Figure 5). These results indicated that bDLE could induce long-term viral inhibition through cell protection, as well as modulate cell susceptibility to viral infection *in vitro*, in agreement with previously reported data on DLE obtained from human donors by molecular methods [9].

## Conclusion

The data presented here were novel in that they proved that bDLE acted by inhibiting HIV-1 infection through protection of the host target CD4 cells at noncytotoxic levels. This effect was found to be modulated through transcriptional factors (NF-κB and SP1) necessary for HIV-1 replication. In addition, bDLE was discovered to act through all viral cycle stages to protect cells from HIV-1 infection for hours without affecting the HIV membrane. Based on our results obtained above, bDLE should be further studied to determine its potential use as a therapeutic agent in HIV-1 infection, especially due to its long-lasting cell protection against HIV-1 infection and lack of negative side effects.

## Methods

### Reagents, cells and HIV-1 isolates

The following reagents were obtained through the AIDS Research and Reference Reagent Program (NIH): HeLa-CD4-LTR-β-gal cells from Dr. Michael Emerman; HL2/3 cells from Dr. Barbara K. Felber and Dr. George N. Pavlakis; HIV-1_IIIB_, fusion inhibitor T-20, integrase inhibitor 118-D-24 and protease inhibitor Amprenavir from Dr. Suzanne Gartner, Dr. Mikulas Popovic and Dr. Robert Gallo. UC781, a no nucleoside reverse-transcriptase inhibitor (NNRTI), was kindly donated by Dr. Gadi Borkow. The bDLE used in our study was produced by the Laboratory of Immunology and Virology at the *Universidad Autonoma de Nuevo Leon*, Mexico, following a modified process described by Lawrence *et al*. [9]. The bDLE was lyophilized, tested for endogenous pyrogens using the Limulus amebocyte lysate assay (MP Biomedicals, Inc.), and determined to be free of bacterial contamination by culturing in media and *in vivo *mice inoculations. The bDLE obtained from 15 × 10^8 ^leukocytes was defined as one unit (1 Unit)[37].

### Cytotoxicity Assays

A stock solution of bDLE was diluted two-fold diluted in growth medium and subsequently added into wells containing 5 × 10^4 ^HeLa-CD4-LTR-β-gal cells. Microtiter plates were incubated at 37°C in a 5% CO_2 _air humidified atmosphere for 24 hours. Assessments of cell viability were carried out using a CellTiter-Glo^® ^Luminescent Cell Viability Assay (Promega). Cytotoxicity was evaluated based on the percentage cell survival relative to the result obtained in the absence of any compound.

### HIV-1 Infection Inhibition Assays

Serial two-fold dilutions of bDLE were mixed with 10^5 ^TCID_50 _of HIV-1_IIIB _and added to the wells containing 5 × 10^4 ^HeLa-CD4-LTR-β-gal cells with a multiplicity of infection (MOI) of 0.2 - 0.5. HIV-1 infection was assessed after 24 hours of incubation by quantifying the activity of the β-galactosidase produced after infection with the Beta-Glo Assay System (Promega). The 50% inhibitory concentration (IC_50_) was defined according to the percentage of infection inhibited relative to the positive control.

### Virucidal Activity Assay

Serial two-fold dilutions of bDLE were added to HIV-1_IIIB _(T tropic virus) and HIV-1_Ba-L _(M Tropic) cell-free virus. After incubation for 5 min at room temperature, the mixtures were centrifuged three times at 10, 000 rpm, the supernatant fluids removed, and the pellets washed three times. The final pellets were resuspended in Dulbecco's Modified Eagle Medium (DMEM) and placed into 96-well plates with HeLa-CD4-LTR-β-gal cells. The cells were incubated in a 5% CO_2 _humidified incubator at 37°C for 24 h. Assessment of HIV-1 infection was made with the Beta-Glo Assay System. The percentage of residual infectivity after bDLE treatment was then calculated with respect to the positive control of untreated virus.

### Cell-based Fusion Assay

HeLa-derived HL2/3 cells (Env cells), which express the HIV-1_HXB2 _Env, Tat, Gag, Rev, and Nef proteins, were co-cultured with HeLa-CD4-LTR-β-gal cells (CD4 cells) at a 1:1 cell density ratio (5 × 10^4 ^cells/well each) for 24 h in the absence or presence of two-fold dilutions of bDLE, UC781, and T-20 in order to examine whether the compounds interfered with the binding process of HIV-1 Env and the CD4 receptor. Also, both HeLa-CD4-LTR-β-gal and HL2/3 cells were exposed to the aforementioned compounds for only 30 minutes and then washed twice to eliminate residual compound before co-cultivating with the other cell line. Upon fusion of both cell lines, the Tat protein from HL2/3 cells activated β-galactosidase (β-gal) indicator gene expression in HeLa-CD4-LTR-β-gal cells [41]. β-gal activity was quantified with the Beta-Glo Assay System (Promega). The percentage of inhibition of HL2/3-HeLa CD4 cell fusion was calculated with respect to the positive control of untreated cells.

### Time of Addition Experiments

HeLa-CD4-LTR-β-gal cells were infected with 10^5 ^TCID_50 _of HIV-1_IIIB _cell-free virus with a 0.2-0.5 MOI. bDLE (2 IU), T-20 (100 μM), UC781 (70 nM), 118-D-24 (120 μM) and Amprenavir (0.1 mM) were then added upon HIV-1 inoculation (time zero) or at various time points post-inoculation. The reference compounds were added at a concentration several times their EC_50 _for infectivity of HIV-1_IIIB_. Infection inhibition was quantified after 24 h by measuring β-gal activity with the Beta-Glo Assay System.

### Cell Protection Assays

HeLa-CD4-LTR-β-gal cells were incubated with bDLE (2 Units) for 30 minutes and subsequently washed with PBS three times. Then, the cells were exposed to 10^5 ^TCID_50 _of HIV-1_IIIB _cell-free virus with a 0.2-0.5 MOI for different times (1, 5, 7, 10, 24 and 48 h). Infection inhibition was quantified after 24 h by measuring β-gal activity with the Beta-Glo Assay System.

### Statistical analysis

Graphs were done with *SigmaPlot 10.0 *software and the values shown are means ± standard deviations from three separate experiments, each of which was carried out in duplicate. Cytotoxicity and inhibition assessment graphs are linear regression curves done with *SigmaPlot *10.0 software.

## Competing interests

The authors declare that they have no competing interests.

## Authors' contributions

All authors read and approved the final manuscript. HHL participated in the conception and experimental design of the *in vitro *HIV-1 manipulation and infection assays, in the analysis and interpretation of the data, and in the writing and revision of this report. LI-T participated in the analysis and interpretation the results. HHL and LI-T made equal contributions to this study. EN-GT participated in the analysis and interpretation of the data and in writing and revising this report. SM-FT participated in the analysis and writing of the report. JIB participated in revising this report. CR-P participated in the experimental design of this research.

## References

[B1] Fauci AS The AIDS epidemic--considerations for the 21st century N Engl J Med 1999 341 1046 1050 10.1056/NEJM199909303411406 10502595

[B2] Ganguly N State of the Globe: The Immunological Quest for an HIV/AIDS Vaccine Continues J Glob Infect Dis 2011 3 209 210 10.4103/0974-777X.83523 21887049 PMC3162804

[B3] Palella FJ Jr Delaney KM Moorman AC Loveless MO Fuhrer J Satten GA Declining morbidity and mortality among patients with advanced human immunodeficiency virus infection. HIV Outpatient Study Investigators N Engl J Med 1998 338 853 860 10.1056/NEJM199803263381301 9516219

[B4] Sterne JA May M Costagliola D de WF Phillips AN Harris R Timing of initiation of antiretroviral therapy in AIDS-free HIV-1-infected patients: a collaborative analysis of 18 HIV cohort studies Lancet 2009 373 1352 1363 19361855 10.1016/S0140-6736(09)60612-7 PMC2670965

[B5] Johnson VA Brun-Vezinet F Clotet B Gunthard HF Kuritzkes DR Pillay D Update of the drug resistance mutations in HIV-1: December 2010 Top HIV Med 2010 18 156 163 21245516

[B6] Lara HH Garza-Trevino EN Ixtepan-Turrent L Singh DK Silver nanoparticles are broad-spectrum bactericidal and virucidal compounds J Nanobiotechnology 2011 9 30 10.1186/1477-3155-9-30 21812950 PMC3199605

[B7] Caffrey M HIV envelope: challenges and opportunities for development of entry inhibitors Trends Microbiol 2011 19 191 197 10.1016/j.tim.2011.02.001 21377881 PMC3071980

[B8] Kirkpatrick CH Transfer factors: identification of conserved sequences in transfer factor molecules Mol Med 2000 6 332 341 10949913 PMC1949950

[B9] Fernandez-Ortega C Dubed M Ruibal O Vilarrubia OL Menendez de San Pedro JC Navea L Inhibition of in vitro HIV infection by dialysable leucocyte extracts Biotherapy 1996 9 33 40 10.1007/BF02628654 8993755

[B10] Lawrence HS Borkowsky W Transfer factor--current status and future prospects Biotherapy 1996 9 1 5 10.1007/BF02628649 8993750

[B11] Franco-Molina MA Mendoza-Gamboa E Castillo-Leon L Tamez-Guerra RS Rodriguez-Padilla C Bovine dialyzable leukocyte extract modulates the nitric oxide and pro-inflammatory cytokine production in lipopolysaccharide-stimulated murine peritoneal macrophages in vitro J Med Food 2005 8 20 26 10.1089/jmf.2005.8.20 15857204

[B12] Franco-Molina MA Mendoza-Gamboa E Castillo-Tello P Isaza-Brando CE Garcia ME Castillo-Leon L Bovine dialyzable leukocyte extract modulates cytokines and nitric oxide production in lipopolysaccharide-stimulated human blood cells Cytotherapy 2007 9 379 385 10.1080/14653240701320262 17573613

[B13] Kirkpatrick CH Rich RR Smith TK Effect of transfer factor on lymphocyte function in anergic patients J Clin Invest 1972 51 2948 2958 10.1172/JCI107119 5080419 PMC292445

[B14] Lang I Nekam K Gergely P Petranyi G Effect in vivo and in vitro treatment with dialyzable leukocyte extracts on human natural killer cell activity Clin Immunol Immunopathol 1982 25 139 144 10.1016/0090-1229(82)90173-8 7151336

[B15] Fudenberg HH Pizza G Transfer factor 1993: new frontiers Prog Drug Res 1994 42 309 400 8085011 10.1007/978-3-0348-7153-2_7

[B16] Lara HH Ixtepan Turrent L Garza Treviño EN Tamez-Guerra RS Rodriguez-Padilla C (Eds) Clinical and Immunological assessment in breast cancer patients receiving anticancer therapy and bovine dialyzable extract as an adjuvant Experimental and Therapeutic Medicine 2010 1 425 431 10.3892/etm_00000066 PMC3445885 22993557

[B17] Estrada-Parra S Nagaya A Serrano E Rodriguez O Santamaria V Ondarza R Comparative study of transfer factor and acyclovir in the treatment of herpes zoster Int J Immunopharmacol 1998 20 521 535 10.1016/S0192-0561(98)00031-9 9839657

[B18] Mazzella G Ronchi M Villanova N Mohamed AA Pizza G De VC Treatment of chronic B virus hepatitis with specific transfer factor Journal Exp Pathol 1987 1 421 423 3454802

[B19] Fabre RA Perez TM Aguilar LD Rangel MJ Estrada-Garcia I Hernandez-Pando R Transfer factors as immunotherapy and supplement of chemotherapy in experimental pulmonary tuberculosis Clin Exp Immunol 2004 136 215 223 10.1111/j.1365-2249.2004.02454.x 15086383 PMC1809022

[B20] Hastings RC Morales MJ Shannon EJ Jacobson RR Preliminary results on the safety and efficacy of transfer factor in leprosy Int J Lepr Other Mycobact Dis 1976 44 275 945240

[B21] Delgado O Romano EL Belfort E Pifano F Scorza JV Rojas Z Dialyzable leukocyte extract therapy in immunodepressed patients with cutaneous leishmaniasis Clin Immunol Immunopathol 1981 19 351 359 10.1016/0090-1229(81)90078-7 7249417

[B22] Louie E Borkowsky W Klesius PH Haynes TB Gordon S Bonk S Treatment of cryptosporidiosis with oral bovine transfer factor Clin Immunol Immunopathol 1987 44 329 334 10.1016/0090-1229(87)90077-8 3621678

[B23] Masi M De VC Baricordi OR Transfer factor in chronic mucocutaneous candidiasis Biotherapy 1996 9 97 103 10.1007/BF02628665 8993766

[B24] Levin AS Spitler LE Stites DP Fudenberg HH Wiskott-Aldrich syndrome, a genetically determined cellular immunologic deficiency: clinical and laboratory responses to therapy with transfer factor Proc Natl Acad Sci USA 1970 67 821 828 10.1073/pnas.67.2.821 5289024 PMC283279

[B25] Wolf RE Fudenberg HH Welch TM Spitler LE Ziff M Treatment of Bechcet's syndrome with transfer factor JAMA 1977 238 869 871 10.1001/jama.238.8.869 577975

[B26] Valdes Sanchez AF Martin Rodriguez OL Lastra AG [Treatment of extrinsic bronchial asthma with transfer factor] Rev Alerg Mex 1993 40 124 131 9312340

[B27] Kaminkova J Lange CF Transfer factor and repeated otitis media Cell Immunol 1984 89 259 264 10.1016/0008-8749(84)90217-X 6333288

[B28] Abramson A Khan A Tate GW Jr Martin RG Hill NO Immunocompetence and transfer factor therapy in uveitis Br J Ophthalmol 1980 64 332 338 10.1136/bjo.64.5.332 7437394 PMC1043690

[B29] Franco-Molina MA Mendoza-Gamboa E Zapata-Benavides P Vera-Garcia ME Castillo-Tello P Garcia dlF IMMUNEPOTENT CRP (bovine dialyzable leukocyte extract) adjuvant immunotherapy: a phase I study in non-small cell lung cancer patients Cytotherapy 2008 10 490 496 10.1080/14653240802165681 18821359

[B30] Whyte RI Schork MA Sloan H Orringer MB Kirsh MM Adjuvant treatment using transfer factor for bronchogenic carcinoma: long-term follow-up Ann Thorac Surg 1992 53 391 396 10.1016/0003-4975(92)90256-4 1540053

[B31] Franco-Molina MA Mendoza-Gamboa E Miranda-Hernandez D Zapata-Benavides P Castillo-Leon L Isaza-Brando C In vitro effects of bovine dialyzable leukocyte extract (bDLE) in cancer cells Cytotherapy 2006 8 408 414 10.1080/14653240600847266 16923617

[B32] Mendoza-Gamboa E Franco-Molina MA Zapata-Benavides P Castillo-Tello P Vera-Garcia ME Tamez-Guerra RS Bovine dialyzable leukocyte extract modulates AP-1 DNA-binding activity and nuclear transcription factor expression in MCF-7 breast cancer cells Cytotherapy 2008 10 212 219 10.1080/14653240801891659 18368600

[B33] Franco-Molina MA Mendoza-Gamboa E Castillo-Leon L Tamez-Guerra RS Rodriguez-Padilla C Bovine dialyzable leukocyte extract protects against LPS-induced, murine endotoxic shock Int Immunopharmacol 2004 4 1577 1586 10.1016/j.intimp.2004.06.014 15454111

[B34] Roberts L Passmore JA Williamson C Little F Bebell LM Mlisana K Plasma cytokine levels during acute HIV-1 infection predict HIV disease progression AIDS 2010 24 819 831 10.1097/QAD.0b013e3283367836 20224308 PMC3001189

[B35] Gottlieb AA Sizemore RC Gottlieb MS Kern CH Rationale and clinical results of using leucocyte-derived immunosupportive therapies in HIV disease Biotherapy 1996 9 27 31 10.1007/BF02628653 8993754

[B36] Raise E Guerra L Viza D Pizza G De VC Schiattone ML Preliminary results in HIV-1-infected patients treated with transfer factor (TF) and zidovudine (ZDV) Biotherapy 1996 9 49 54 10.1007/BF02628656 8993757

[B37] McMeeking A Borkowsky W Klesius PH Bonk S Holzman RS Lawrence HS A controlled trial of bovine dialyzable leukocyte extract for cryptosporidiosis in patients with AIDS J Infect Dis 1990 161 108 112 10.1093/infdis/161.1.108 2404072

[B38] Pizza G Chiodo F Colangeli V Gritti F Raise E Fudenberg HH Preliminary observations using HIV-specific transfer factor in AIDS Biotherapy 1996 9 41 47 10.1007/BF02628655 8993756

[B39] Li JC Yim HC Lau AS Role of HIV-1 Tat in AIDS pathogenesis: its effects on cytokine dysregulation and contributions to the pathogenesis of opportunistic infection AIDS 2010 24 1609 1623 10.1097/QAD.0b013e32833ac6a0 20588103

[B40] Lim SP Garzino-Demo A The human immunodeficiency virus type 1 Tat protein up-regulates the promoter activity of the beta-chemokine monocyte chemoattractant protein 1 in the human astrocytoma cell line U-87 MG: role of SP-1, AP-1, and NF-kappaB consensus sites J Virol 2000 74 1632 1640 10.1128/JVI.74.4.1632-1640.2000 10644332 PMC111637

[B41] Lalonde MS Lobritz MA Ratcliff A Chamanian M Athanassiou Z Tyagi M Inhibition of both HIV-1 reverse transcription and gene expression by a cyclic peptide that binds the Tat-transactivating response element (TAR) RNA PLoS Pathog 2011 7 e1002038 10.1371/journal.ppat.1002038 21625572 PMC3098202

[B42] Fernandez-Ortega C Dubed M Ramos Y Navea L Alvarez G Lobaina L Non-induced leukocyte extract reduces HIV replication and TNF secretion Biochem Biophys Res Commun 2004 325 1075 1081 10.1016/j.bbrc.2004.10.142 15541398

[B43] Ojeda MO Fernandez-Ortega C Rosainz MJ Dialyzable leukocyte extract suppresses the activity of essential transcription factors for HIV-1 gene expression in unstimulated MT-4 cells Biochem Biophys Res Commun 2000 273 1099 1103 10.1006/bbrc.2000.3065 10891378

[B44] Ojeda MO van't Veer C Fernandez Ortega CB Arana Rosainz MJ Buurman WA Dialyzable leukocyte extract differentially regulates the production of TNFalpha, IL-6, and IL-8 in bacterial component-activated leukocytes and endothelial cells Inflamm Res 2005 54 74 81 10.1007/s00011-004-1326-5 15750714

[B45] Elrefaei M Burke CM Baker CA Jones NG Bousheri S Bangsberg DR HIV-specific TGF-beta-positive CD4+ T cells do not express regulatory surface markers and are regulated by CTLA-4 AIDS Res Hum Retroviruses 2010 26 329 337 10.1089/aid.2009.0149 20433405 PMC2933167

[B46] Hiscott J Kwon H Genin P Hostile takeovers: viral appropriation of the NF-kappaB pathway J Clin Invest 2001 107 143 151 10.1172/JCI11918 11160127 PMC199181

[B47] Carey JT Lederman MM Treatment of AIDS with transfer factor JAMA 1987 258 3515 3516 3682152 10.1001/jama.1987.03400240047018

[B48] Flory E Weber CK Chen P Hoffmeyer A Jassoy C Rapp UR Plasma membrane-targeted Raf kinase activates NF-kappaB and human immunodeficiency virus type 1 replication in T lymphocytes J Virol 1998 72 2788 2794 9525598 10.1128/jvi.72.4.2788-2794.1998 PMC109723

[B49] Alvarez-Thull L Kirkpatrick CH Profiles of cytokine production in recipients of transfer factors Biotherapy 1996 9 55 59 10.1007/BF02628657 8993758

[B50] Lara HH Ayala-Nunez NV Ixtepan-Turrent L Rodriguez-Padilla C Mode of antiviral action of silver nanoparticles against HIV-1 J Nanobiotechnology 2010 8 1 10.1186/1477-3155-8-1 20145735 PMC2818642

[B51] Yang QE Stephen AG Adelsberger JW Roberts PE Zhu W Currens MJ Discovery of small-molecule human immunodeficiency virus type 1 entry inhibitors that target the gp120-binding domain of CD4 J Virol 2005 79 6122 6133 10.1128/JVI.79.10.6122-6133.2005 15857997 PMC1091715

[B52] Jones PL Korte T Blumenthal R Conformational changes in cell surface HIV-1 envelope glycoproteins are triggered by cooperation between cell surface CD4 and co-receptors J Biol Chem 1998 273 404 409 10.1074/jbc.273.1.404 9417096

[B53] Demarchi F d'Adda di FF Falaschi A Giacca M Activation of transcription factor NF-kappaB by the Tat protein of human immunodeficiency virus type 1 J Virol 1996 70 4427 4437 8676466 10.1128/jvi.70.7.4427-4437.1996 PMC190376

[B54] Gaynor R Cellular transcription factors involved in the regulation of HIV-1 gene expression AIDS 1992 6 347 363 10.1097/00002030-199204000-00001 1616633

[B55] Auwerx J Stevens M Van Rompay AR Bird LE Ren J De CE The phenylmethylthiazolylthiourea nonnucleoside reverse transcriptase (RT) inhibitor MSK-076 selects for a resistance mutation in the active site of human immunodeficiency virus type 2 RT J Virol 2004 78 7427 7437 10.1128/JVI.78.14.7427-7437.2004 15220416 PMC434123

[B56] Hombrouck A Van RB Michiels M Noppe W Christ F Eneroth A Preclinical evaluation of 1H-benzylindole derivatives as novel human immunodeficiency virus integrase strand transfer inhibitors Antimicrob Agents Chemother 2008 52 2861 2869 10.1128/AAC.00210-08 18541726 PMC2493095

[B57] Stevens M Pannecouque C De CE Balzarini J Novel human immunodeficiency virus (HIV) inhibitors that have a dual mode of anti-HIV action Antimicrob Agents Chemother 2003 47 3109 3116 10.1128/AAC.47.10.3109-3116.2003 14506017 PMC201129

[B58] Svarovskaia ES Barr R Zhang X Pais GC Marchand C Pommier Y Azido-containing diketo acid derivatives inhibit human immunodeficiency virus type 1 integrase in vivo and influence the frequency of deletions at two-long-terminal-repeat-circle junctions J Virol 2004 78 3210 3222 10.1128/JVI.78.7.3210-3222.2004 15016842 PMC371038

[B59] Witvrouw M Balzarini J Pannecouque C Jhaumeer-Laulloo S Este JA Schols D SRR-SB3, a disulfide-containing macrolide that inhibits a late stage of the replicative cycle of human immunodeficiency virus Antimicrob Agents Chemother 1997 41 262 268 9021177 10.1128/aac.41.2.262 PMC163699

[B60] Witvrouw M Fikkert V Pluymers W Matthews B Mardel K Schols D Polyanionic (i.e., polysulfonate) dendrimers can inhibit the replication of human immunodeficiency virus by interfering with both virus adsorption and later steps (reverse transcriptase/integrase) in the virus replicative cycle Mol Pharmacol 2000 58 1100 1108 11040059

[B61] Zhang X Pais GC Svarovskaia ES Marchand C Johnson AA Karki RG Azido-containing aryl beta-diketo acid HIV-1 integrase inhibitors Bioorg Med Chem Lett 2003 13 1215 1219 10.1016/S0960-894X(03)00059-3 12643946

[B62] Corallini A Sampaolesi R Possati L Merlin M Bagnarelli P Piola C Inhibition of HIV-1 Tat activity correlates with down-regulation of bcl-2 and results in reduction of angiogenesis and oncogenicity Virology 2002 299 1 7 10.1006/viro.2002.1459 12167335

[B63] Duh EJ Maury WJ Folks TM Fauci AS Rabson AB Tumor necrosis factor alpha activates human immunodeficiency virus type 1 through induction of nuclear factor binding to the NF-kappa B sites in the long terminal repeat Proc Natl Acad Sci USA 1989 86 5974 5978 10.1073/pnas.86.15.5974 2762307 PMC297754

[B64] Zussman A Lara L Lara HH Bentwich Z Borkow G Blocking of cell-free and cell-associated HIV-1 transmission through human cervix organ culture with UC781 AIDS 2003 17 653 661 10.1097/00002030-200303280-00002 12646787

